# Supplementary material for: Identification of Freezing-Responsive microRNAs and Their Targets in Chinese Jujube by Small RNA and Degradome Sequencing
Source: Int J Mol Sci. 2026 Sep 4;27(17):7912. doi: 10.3390/ijms27177912 (PMC13566890; doi:10.3390/ijms27177912)
Supplement: Supplementary file 1 [file ijms-27-07912-s001.zip › Supplementary Material S2/novel_miR_202.pdf]

Provisional ID	: novel_miR_202
Score total	: 38.2
Score for star read(s)	: 3.9
Score for read counts	: 36.3
Score for mfe	: 0.1
Score for randfold	: -2.2
Score for cons. seed	:
Total read count	: 83
Mature read count	: 80
Loop read count	: 0
Star read count	: 3

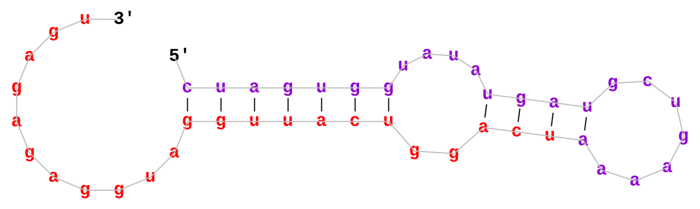

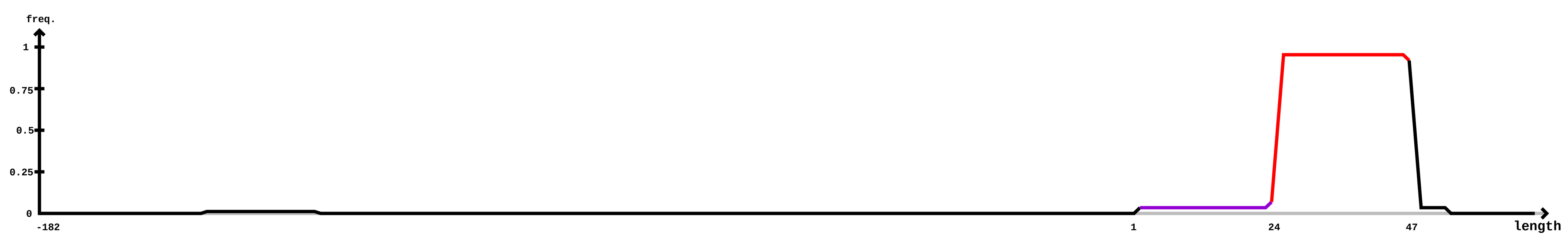

Star Mature

```
5'- guguuauaaaauuggaggcauuuugaaguauaacuuucuggaacuagauauauagauugauuggggauaauuuuuaucgaaagaauggaguagaugaaauauuuuugguguguuucuguauguaauuuuauuuauugacuucuuaauuguuuccaauugcagacauggugcuugcaaauaagcuagugguauaugaugcugaaaaucaggucauuggauggagagaguacaacuguaaguaucuuugauu -3' obs
    guguuauaaaauuggaggcauuuugaaguauaacuuucuggaacuagauauauagauugauuggggauaauuuuuaucgaaagaauggaguagaugaaauauuuuugguguguuucuguauguaauuuuauuuauugacuucuuaauuguuuccaauugcagacauggugcuugcaaauaagcuagugguauaugaugcugaaaaucaggucauuggauggagagaguacaacuguaaguaucuuugauu     exp
    (((((....((((((((((((..(((((.........(((((....(((((((((...((((((((((..(((((((..............))))))))))))))))).....)))))))))..))))).......)))))..)).))))))))))))...)))))(((((((((.....(((..(.((..(((((.((((...)))))))))...)).)....))).....))))))))).......   reads   mm   sample
    ..........................aAuauaacuuucuggaacu...................................................................................................................................................................................   1   1   JCK-1
    .............................................................................................................................................................................cuagugguauaugaugcugaaaa..........................................   3   0   LT-2
    .......................................................................................................................................................................................................auUaggucauuggauggagagag.......................   3   1   LT-2
    ........................................................................................................................................................................................................ucaggucauuggauggagagagC.......................   1   1   LCK-2
    ........................................................................................................................................................................................................ucaggucauuggauggagagagu.......................   36   0   LCK-1
    ........................................................................................................................................................................................................ucaggucauuggauggagagagu.......................   40   0   LCK-2
    .........................................................................................................................................................................................................caggucauuggauggagagaguacaacu................   2   0   LCK-3
    .........................................................................................................................................................................................................caggucauuggauggagagaguacaacu................   1   0   JCK-2
```
